# Current News and Opinion

**Published:** 1883-06

**Authors:** 


### Current News and Opinion.

### EDUCATIONAL ASSOCIATION.

In 1866 the faculties of the different dental colleges of this country formed an association, the object of which was to promote the interests of dental education. The organization was effected with good promise for the future. It continued for two or three years, working harmoniously, and accomplishing something each year for dental education. At the third meeting one college withdrew without even intimating a reason therefor ; then another college in the same city seemed impelled to take the same course, then one in a neighboring city for self-protection did the same. As a result the association was abandoned for the time at least. From that to the present, no effort has been made for its revival. There were then six colleges in the United States; now there are about twenty. Certainly, if when there were six such an association was desirable, now that there are twenty it is more desirable and important. It has been suggested by the representatives of several of the colleges that a meeting be held at Niagara, at the time of the meeting of the American Dental Association, with the view of reviving the Association, or forming a new one. This would certainly be a step in the right direction, and it seems eminently desirable that such a movement be made. Let all who are interested consider the matter, and in some way give expression to their opinions and conclusions in the matter.—*Dental Register.*

---

### THE ODONTOLOGICAL SOCIETY.

The next meeting of the New York Odontological Society will be held at the country residence of Dr. C. E. Francis, in Glenbrook, Conn., Tuesday afternoon, June 19, 1883. It is hoped that as many members as possible will make it convenient to attend, and that they will so notify Dr. Francis at 33 West 47th Street, New York. Members will take the three o'clock New Haven train at the Grand Central Depot, and purchase return tickets for Glenbrook.

J. Morgan Howe,

Corresponding Secretary.

---

### PENNSYLVANIA STATE BOARD OF DENTAL EXAMINERS.

The Pennsylvania Board of Examiners will, as usual, hold meetings for the examination of candidates during the session of the State Dental Society, commencing Tuesday, July 31, 1883, and continuing three days, at Cresson, Pennsylvania. Applicants for certificates will be required to show specimens of work in both operative and mechanical departments.

W. E. MAGILL,
Chairman Ex. Com.,
Erie, Pa.

---

### DETROIT DENTAL SOCIETY.

The Detroit Dental Society has elected the following officers for the ensuing year:

President—Dr. George L. Field.

Vice-President—Dr. Henry Cowie.

Treasurer—Dr. James Cleland.

Secretary—Dr. H. K. Lathrop.

Member of the Board of Censors—Dr. E. C. Moore.

The society, which has been in existence for one year, has about twenty members, and is in a good condition.

---

### INDIANA STATE DENTAL ASSOCIATION.

The twenty-fifth anniversary of the Indiana State Dental Association will be held this year in Indianapolis, in the parlors of the new Dennison House, beginning Tuesday, June 26, at 11 o'clock A. M. As this is a quarter centennial, the list of subjects used in 1858, when the Association was organized, will be used again in June, viz:

1st. Best means of preserving teeth.

2d. Treatment of exposed nerves.

3d. The best means to correct irregularities.

4th. Mechanical dentistry.

5th. Miscellaneous subjects.

Those attending the meeting should stop at the Dennison House, where they will be accommodated at $2.50 per day. The various dealers and manufacturers will exhibit at the Dennison. This quarter centennial celebration will be closed with a jubilee and banquet.

J. E. CRAVENS,

President.

---

### A SINGULAR STATEMENT.

At the last meeting of the Brooklyn Dental Society, Dr. Thomas, of the S. S. White Manufacturing Company, said that in his experience of a number of years in several of the largest dental depots in the country, he had observed that at least one-third more left incisor and cuspid teeth were sold than the corresponding ones on the right side of the mouth, indicating that the natural anterior teeth upon the left side were lost more rapidly than those on the right. He said that sets of the six front teeth were continually broken up to supply this demand, and that dealers always had a quantity of the odd teeth left.

The statement caused considerable surprise to the members present, who would be glad of further information.

---

### THE A. M. A.

At the late meeting of the American Medical Association, Prof. T. W. Brophy, of Chicago, was elected chairman of the section of Oral and Dental Surgery. Dr. J. S. Marshall, of Illinois, was elected Secretary.

Dr. Brophy was largely instrumental in the establishment of this section, and has always been active in its support. His election as Chairman was, therefore, but a proper recognition of his labors, as well as a tribute to his unquestioned ability and merit.

